# Effects of health education on spousal knowledge and participation in birth preparedness in Farafenni Regional Hospital, The Gambia: a randomized trial

**DOI:** 10.1186/s12884-021-03605-y

**Published:** 2021-02-12

**Authors:** Haddy Tunkara- Bah, Florence O. Adeyemo, Friday E. Okonofua

**Affiliations:** 1 Department of Nursing, University of The Gambia, Serrekunda, Gambia; 2 Department of Nursing, University of Benin, Benin City, Nigeria; 3 Department of Obstetrics and Gynae, University of Benin, Benin City, Nigeria

**Keywords:** Health education, Intervention, Birth preparedness, Spouses, Farafenni, The Gambia

## Abstract

**Background:**

The Gambia is a male-dominant society in which the cultural norms empower husbands to decide when and where their wives seek care, yet they are not always involved in maternal health care services. Therefore, the purpose of this study was to design and measure the effects of antenatal health education on spousal participation in birth preparedness in Farafenni and satellite villages.

**Methods:**

The study used a quasi-experimental design, and the participants were 300 spouses of pregnant women attending their antenatal care booking at Farafenni Hospital. A multistage sampling method was used to select the study participants who were then equally distributed to the intervention and comparison groups. Pre-test data were collected from both groups. Thereafter, the intervention group was exposed to two health education sessions on obstetric danger signs and birth preparedness. The post-test data were collected immediately before discharge of the participants’ wives after institutional delivery or within 2 weeks post-delivery for those who did not accompany their wives to the health care institution, or whose wives delivered at home. IBM SPSS version 21 software was used to analyze the data.

**Results:**

The differences between the demographic characteristics of participants in the intervention and comparison groups were not statistically significant except for the highest level of education achieved. After controlling for the demographic variables, the health education administered to the intervention group effectively increased knowledge on birth preparedness among them (F (1, 255) = 376.108, *p* < .001). Every unit increase in the intervention led to a unit increase in the spouses’ knowledge on birth preparedness (β = 0.789, *p* <  0.001). Furthermore, the participants in the intervention group had higher mean score (M = 4.4; SD = 0.8) on participation in birth preparedness than those in the comparison group (M = 0.9; SD = 0.8). The spouses in the intervention group were four times more likely to be prepared for the delivery of their wives after being exposed to the health education than those in the comparison group (F (1, 255) = 522.414, *p* < .001).

**Conclusion:**

The study provides evidence that educating men on maternal health care can improve their level of participation in birth preparedness.

**Trial registration:**

**Name of Registry:** Pan African Clinical Trial Registry (www.pactr.org). **Registry Number:** PACTR202004752273171. **Date of Registration:** 19th April 2020. Retrospectively Registered.

**Supplementary Information:**

The online version contains supplementary material available at 10.1186/s12884-021-03605-y.

## Introduction

### Background

The high number of women dying during pregnancy, delivery, and post-partum periods remains a significant public health problem. The global maternal mortality ratio (MMR) in 2017 was estimated at 211/100,000 live births and the majority of them occurred in developing countries [1]. Regionally, sub-Saharan Africa alone accounted for more than half of the global maternal mortalities [2]. The Gambia was classified among the 18 sub-Saharan Africa countries with a high maternal mortality ratio (MMR) at 597/100,000 live births in 2017 [2]. Farafenni is located in the rural Gambia, and the National Demographic Health Survey of 2019 indicated that this area’s maternal mortality ratio (MMR) is two-fold higher than that of the urban parts of the country.

Despite The Gambia being a male-dominant society in which the cultural norms empower husbands to decide when and where their wives seek health care, yet they are not always involved in maternal health care services. The persistent poor birth outcomes among pregnant women in The Gambia could be due to the non-use of available modern health services such as health facility-based delivery and family planning services, and a lack of birth preparedness by a sizeable proportion of rural women [3]. The National Demographic Health Survey of 2019 reported that the institutional delivery rate in The Gambia was 84% and was lower in the rural areas, estimated at 75% [3]. According to Lerberg, et al., 72% of the pregnant women in the North Bank East Region of The Gambia (where Farafenni is located) reported that they delivered under the care of traditional birth attendants (TBAs) who perform deliveries outside health facilities, lack the skill required for early identification and management of danger signs of pregnancy and delivery [4]. However, most maternal mortalities occur during labour, childbirth, and up to 24 h after delivery, hence the need for quality care during this period [5, 6].

The poor utilization of skilled delivery services in The Gambia may be a result of that pregnancy and childbirth continue to be viewed solely as women issues [7, 8]. Spousal participation in antenatal health education on birth preparedness may be an effective strategy in the crusade against poor pregnancy outcomes, especially in patriarchal societies [9] like The Gambia. Birth preparedness is about knowing obstetric danger signs, preparedness for a childbirth, knowledge of which health facility to go to in case of an emergency, saving money for transportation and other needs, donating blood and save it for future use, and temporary family care in case of emergencies. However, men who take responsibility for economic choices and decision-making may not always possess the required knowledge for early identification of obstetrics danger signs [10]. This lack of knowledge can lead to delay in the decision to seek expert obstetric care among women and thus contributes to poor outcomes in maternal health care. It was reported among Gambian women that factors contributing to home deliveries with unskilled birth attendance included a delay in securing transport and lack of money [11]. Transportation to health facilities sometimes becomes a challenge for many rural residents in The Gambia especially during the rainy season due to long-distance and poor road networks. Many rural residents in this country also depend on farming and only have money after selling their farm products. Therefore, to overcome the problem of delay in securing transport and promote institutional deliveries among rural Gambian women, men who hold the economic powers should be taught the importance of saving money and identifying means of transport in preparation for the deliveries of their wives. Male involvement in birth preparedness enables them to support their spouses in seeking professional health care and to avoid delays in the decision making process [12].

Delivery is also viewed as an unclean process due to the bleeding and drainage of amniotic fluid. Women in labour usually come to the delivery rooms with old and unwanted clothing which are sometimes even unclean [11]. This may be a contributing factor to the high rate of neonatal and puerperal sepsis in The Gambia [13]. Since in most cases, men are the providers of non-medical delivery care requirements in The Gambia, raising their awareness on birth preparedness will help to ensure the availability of materials for a clean delivery.

Furthermore, information, education, and communication during antenatal care are poor [14] and only 27% of the pregnant women from Farafenni and satellite villages reported receiving information on birth preparedness during antenatal care [15]. Similar poor communication was also reported in a study on social and cultural barriers to husbands’ involvement in maternal health in rural Gambia where the majority of participants reported that they were usually not aware of their wives’ pregnancy until it reached a rather late stage [15]. Most of the studies conducted on male partners’ involvement in maternal care in The Gambia were descriptive studies that were restricted to describing the characteristics of male partners’ involvement.

### General objective

The main objective of this study was to measure the effects of antenatal health education intervention on spousal knowledge and participation in birth preparedness in Farafenni and satellite villages.

## Methods

### Trial design

**Fig. 1:**
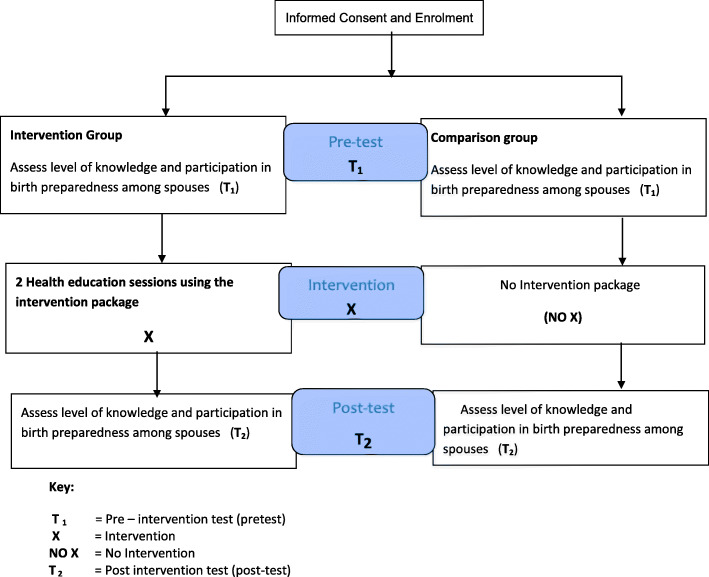
Quasi-experimental design with pre-test and post-test comparison groups

This study used a randomized control trial with pre-test and post-test comparison groups design to evaluate the effect of a health education intervention programme on spousal participation in birth preparedness in Farafenni and satellite villages. This study design was guided by the action points identified during baseline research done among the target group [16]. The study design is simplified in Fig. 1.

### Population of the study

The study population was spouses of pregnant women attending antenatal care at the Maternal and Child Clinic of Farafenni during the data collection period. The participants’ inclusion criteria for this study were as follow:

1. Spouses of pregnant women attending their antenatal care (ANC) booking at the Maternal and Child Clinic, Farafenni, with a gestational age between 16 to 28 weeks.
2. The spouses who came from the villages and Farafenni Wards identified as study sites and planning to stay in the area for at least 1 month post-delivery of their wives.
3. Living with their wives and expected to be present during the pregnancy and delivery periods.
4. Ability to speak Fula, Wollof, Mandinka, or English language.
5. Permission must be given by their pregnant wives to contact them and for their participation. This is because women should be given the autonomy to choose whether their husbands can be involved in their maternal health care or not. According, WHO, despite the benefits of male partner participation in maternal health care, it should never be a requirement for women to access skilled care thus women should be empowered to make such a decision [6].

The exclusion criteria were:

1. Spouses who met the inclusion criteria but were less than 18 years of age.
2. Health-care professionals or studied medical sciences
3. Spouses who were sick or traveled during the time of study participants’ enrollment period.

### Study setting

This study was conducted at the Antenatal Clinic of the Farafenni Regional Hospital for easy identification and access to the target group. Farafenni is located in the North Bank East Region of The Gambia and it has both urban and rural characteristics, as well as diverse ethnic distribution. Therefore, the findings obtained from this study can give a picture of both rural and urban communities in The Gambia. The health education sessions were conducted in two separate rooms, i.e., the Antenatal Examination and the Weighing Rooms, as these rooms were not in use during the intervention days.

### Intervention

This study was a clinic-based intervention. There was no antenatal care (ANC) on Fridays in Farafenni Antenatal Clinic, so these days were chosen as research days. The health education intervention and comparison placebo were conducted on alternate Fridays. This had helped to prevent the two groups from meeting in the clinic, thereby reducing the risk of sharing the interventional messages with the comparison group. Thirty (30) spouses were invited each Friday and were requested to come without their wives but with their antenatal cards. The duration of the intervention lasted for six (6) months.

There was no prior antenatal health education programme for spouses of pregnant women at Farafenni. Therefore, the health education curriculum was designed by the researcher using the indicators of monitoring birth preparedness and complication readiness for maternal and newborn health developed by Jhpiego [12] and the research objectives as guides. The health education curriculum was divided into two modules. Module one included information on birth preparedness (including danger signs of pregnancy and childbirth) while module two covered normal signs of labour and institutional delivery care. Besides, detailed health education posters that reviewed the main messages of the modules were used as visual aids to enhance learning during the intervention sessions. Posters with danger signs of pregnancy and childbirth and birth preparedness indicators were adopted and used in the health education sessions. Physical samples of the materials needed for a clean delivery were also made available during the sessions. With the assistance of persons knowledgeable in the local culture, the health education modules and visual aids were designed to reflect the community’s characteristics and tradition.

The health education sessions were conducted by two research assistants (male and female) and the researchers using Wollof, Mandinka, or Fula local languages depending on the participants’ preferences. The health education was delivered individually (one-to-one) to all the members of the intervention group. There were two health education sessions for each of the spouses in the intervention group as outlined below and summarized in Table 1.

**Table 1:** Health education on birth preparedness and institutional delivery for spouses in the intervention group

Sessions 1	Sessions 2
Occurred immediately after the pre-test	Occurred at 36 weeks of wives’ gestational age
150 spouse attended and delivered one–to-one	150 spouse attended and delivered one–to-one
Recognition of danger signs of pregnancy and delivery and actions to take	Main messages on danger signs of pregnancy and delivery and birth preparedness reviewed
Birth preparedness and complication readiness	Signs of normal labour
All spouses counseled and send to donate blood (for complication readiness)	Importance of institutional delivery and spousal participation
Summary of main messages and poster on danger signs of pregnancy and delivery given to each spouse to take home	Assessed for birth preparedness, if not prepared, spouses were counseled again
Spouses were informed on the expected date of deliveries of their wives and when to come back for the second session	Blood donation was assessed and spouses that did not donate blood were counseled and send for blood donation again

### First health education sessions

One hundred and fifty (150) spouses came for the first health education sessions, and each received 25-min one-to-one health education on obstetric danger signs and birth preparedness. Posters with obstetric danger signs and birth preparedness indicators were also shown to the participants during the health talk. Counseling on blood donation was also conducted during the first health education contact with each spouse as blood donation is part of the birth preparedness process. The spouses who consented to donate blood were referred to donate blood to save for emergency during the pregnancy or delivery periods of their wives. Participants were made to understand that the blood donated may be used for other patients if their wives did not need it to avoid expiration, but the Blood Bank Unit would ensure that blood is made available to them when it is needed. The spouses were also informed of the expected dates of delivery of their wives so that they could make plans for it.

After evaluating their comprehension and reviewing the main messages, each participant was given a typed list of all the materials required for a clean delivery and A-4 size paper photocopies of the posters with the obstetric danger signs and birth preparedness indicator to take home. Each spouse was also informed on the date of his second session, which was at 36 weeks gestation of his wife. Participants’ contact details and expected dates of delivery of their wives were recorded. These records were reviewed at the end of each intervention day and the participants whose follow-up dates (second health education sessions) were due in the next scheduled date for the intervention group were reminded through phone calls or through letters given to their wives during their normal ANC visits to deliver to them. A record of those who donated blood, and that of their wives were kept in the Blood Donation Unit.

### Second health education sessions

A total of 150 participants came for the second session and each was given another 25-min health education on signs of normal labour and the importance of institutional delivery care. These topics constituted module two of the health education package. These sessions started with reviewing the main messages of the first health education session. Thereafter, spouses were taught signs of normal labour to equip them with the required knowledge needed for them to make early decisions for their wives to seek skilled care when they were in labour. Inquiries were made regarding their level of preparation for the deliveries of their wives and blood donation. Those who reported that they did not donate blood were counseled again and referred to the blood donation unit. They were also informed that the post-test assessments would be conducted immediately after the deliveries of their wives. The health educators summarized the health information on each of the topics co2vered into talking points as follows:

- Life-threatening conditions can occur during pregnancy and childbirth.
- Obstetric emergencies are unpredictable.
- Every birth should be planned.
- Early identification of obstetric danger signs and birth preparedness prevent delay in seeking skilled obstetric care.

### Placebo activities for comparison group

**Fig. 2:**
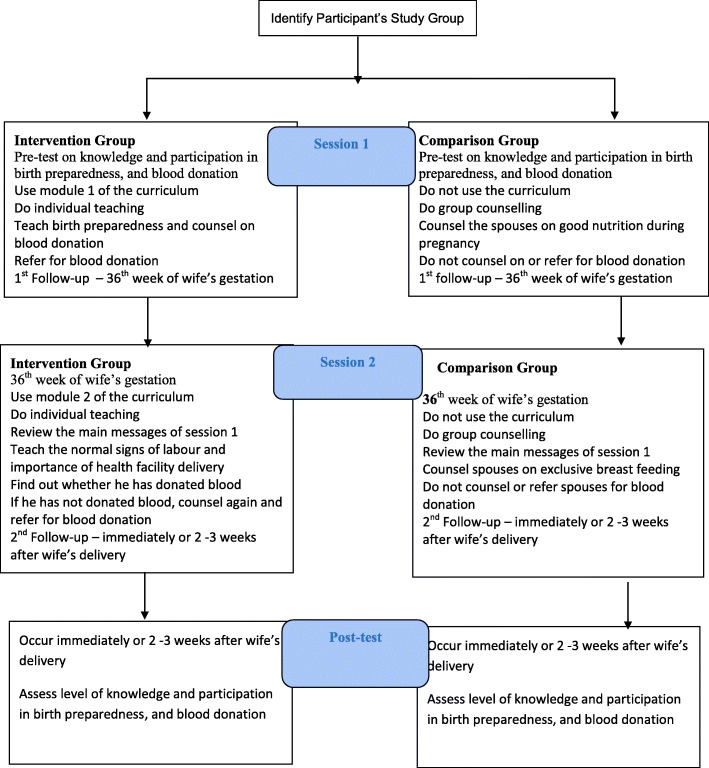
Study Intervention

The treatment of the participants in the comparison group was similar to that of the intervention group except that they did not receive the health education messages from the intervention package but were given group counseling on nutrition during pregnancy in the first session and exclusive breastfeeding in the second (duration: 45 min each). To avoid confusion, this group of participants was informed before the pre-test that the questions that were asked in both the pre-test and post-test were not related to the topics that were discussed in the counseling sessions. The participants from this group were not counseled or sent for blood donation. They were informed that their post-test data would be collected immediately after the deliveries of their wives (Fig. 2).

### Pre-intervention activities

Two buy-in meetings regarding the aims and objectives of the study were undertaken with the Management of Farafenni Regional Hospital in September 2017 before the commencement of the study. Two types of training were also conducted for the staff of the antenatal clinic, labour ward, and blood bank before the starting of the study intervention. The whole site training was given to all the staff including support staff such as general assistants and clerks. The training aimed to familiarize staff about the project, address any concerns, and identify interested staff to serve as research assistants. This training lasted for 45 min. The second component of the training was more intensive and aimed at the research assistants only. Six Community Health Nurses and Midwives were selected as research assistants. Four of the research assistants were from the antenatal clinic whilst the remaining two were from the labour ward. They were given a 90-min refresher training on a variety of topics including obstetric danger signs, preparation for delivery, involving men in maternal care, basic teaching and counseling techniques, and on the structured intervention protocol.

### Study outcomes

The primary outcomes were spousal knowledge of and participation in the birth preparedness of their wives. The Jhpiego prototype questionnaire for monitoring birth preparedness and complication readiness for safe motherhood [12] was adapted and modified to suit the research objectives and target group (Appendix A: Interview Guide). The questionnaire measured knowledge of birth preparedness by asking the participants questions about the danger signs of pregnancy and childbirth, and resources needed when preparing for childbirth. There were seven items under the sections measuring knowledge. A correct response for each item was scored 1 and the wrong response 0. The percentage of the total score for each participant was calculated. A total percentage score between 0 and 30 was defined as low, 31–61 was defined as moderate and 62–100 was defined as a high level of knowledge of birth preparedness.

Spousal level of participation in birth preparedness was measured based on the number of arrangements a spouse had made, including (1) acquired the required materials for clean delivery (included, two clean gowns for mother, two clean pants for mother, a new packet of a pad for mother, two clean buckets with lids, surgical spirit for cleaning baby’s the umbilical stump and a clean wrapper for the baby), (2) saved money for the delivery, (3) donated blood to the blood bank for an emergency, (4) arranged for transportation to the delivery center, (5) made an emergency plan and (6) identified a health facility. Spouses were considered ‘highly’ prepared if more than 3 of these arrangements were reported, moderately prepared if 2–3, and if 0–1 the arrangement was defined as poorly prepared for the delivery of wife [12].

Data on the primary outcomes were collected using a research assistant-administered questionnaire method. The study questionnaire was translated from English to three of the major local languages of the community (Wollof, Mandinka, and Fulla), with the help of an expert in language translation. To ensure quality, before the data collection, six community health nurses and midwives fluent in the three major local languages were selected as research assistants and trained in the administration of the questionnaire. Each data collector was given a sheet containing the basic field protocol. There were two teams of data collectors; one was at the antenatal clinic (with four research assistants) and the other in the labour ward with two research assistants. In each team, one person from the data collectors served as a supervisor. The principal investigators monitored and supervised the overall study, to ensure that the research team adhered to the research procedures.

The pre-test data were collected from the participants immediately after signing/thumb printing the voluntary informed consent forms. The same questionnaire was used to collect both pre-test and post-test data. The participants’ contact details, expected date of delivery of wife, and follow-up appointment dates were noted.

The first follow-up of the participants was at 36 weeks of pregnancy of their wives during which no data was collected. The second follow-ups were used to collect the post-test data. These follow-ups were conducted in the labour and postnatal wards, immediately before discharge of the participants’ wives after delivery by the two research assistants working in the labour ward. The post-test data of those who did not accompany their wives to the health facility for delivery, or those whose wives delivered at home, were collected within two to 3 weeks after the delivery of their wives. To minimize loss to follow-up, participants who did not turn up after an invitation were contacted through phone calls or home visits.

The completed forms from the field were reviewed daily and on-the-spot feedback was provided, with follow-up/callback undertaken, where needed. The data of pre-tested participants who did not participate in the post-test were not included in the analysis to eliminate attrition bias.

### Sample size determination

The estimated sample size was 147 participants in each group (making a total of 294 participants). This was based on the assumption of detecting a 15% effect from the proportion of men accompanying their spouses to antenatal care 20% (13), considering the power of 80% with 5% significant level, a design effect of 1.5, and a non-response rate of 10%. However, this was increased to 150 participants in each group, making a total of 300 participants in this study.

### Randomization

A multistage sampling method was employed to select a representative sample as shown:

#### *Stage 1:* selection of villages and wards

Farafenni Regional Hospital’s Maternal and Child Health Care Catchment Area was divided into rural and urban areas. The rural area was further divided into 12 villages, namely, Macca Farafenni, Yallal Ba, Dutabullul, Gigimarr, Jerri Kaw, Sagab, Kerr Sulay, Kunjo, Elliyasa, Kerr Madi, Kohen Bereto, and Sabach Nyen. The names of these 12 villages were written on pieces of paper, folded, put in a container which was closed and shaken several times to ensure a good mix or randomization of the pieces of paper. Six of these pieces of paper with the names of different villages were randomly withdrawn from the container using the balloting by replacement method. These six selected villages were Macca Farafenni, Dutabullul, Yallal Ba, Kunjo, Kohen Bereto, and Sabach Nyen.

The urban area, which is Farafenni, was divided into four wards and two of them, namely, Farafenni Mauritani, and Farafenni Ballangharr, were selected using random sampling as in the selection of the villages.

#### *Stage 2:* selection of intervention and comparison groups’ residential areas

Selected villages and wards were divided into intervention and comparison groups’ residential areas using random sampling with replacement method to ensure that there were an urban resident and three villages for each of the study groups. This was done to ensure that the participants in the intervention and comparison groups came from the similar environment and had similar characteristics. To ensure that the intervention and comparison groups’ residents were not too near to each other (to reduce the risk of social contacts between the members of the two groups), the balloting was done in such a way that there was always a non-study resident between an intervention and a comparison groups’ residential areas. Farafenni Mauritani, Yalla Ba, Kunjo, and Sabach Nyen were the intervention group’s residents whilst Farafenn Ballangharr, Macca Farafenni, Dutabullul, and Kohen Bereto were that of the comparison group.

#### *Stage 3:* selection of participants for intervention and comparison groups

**Fig. 3:**
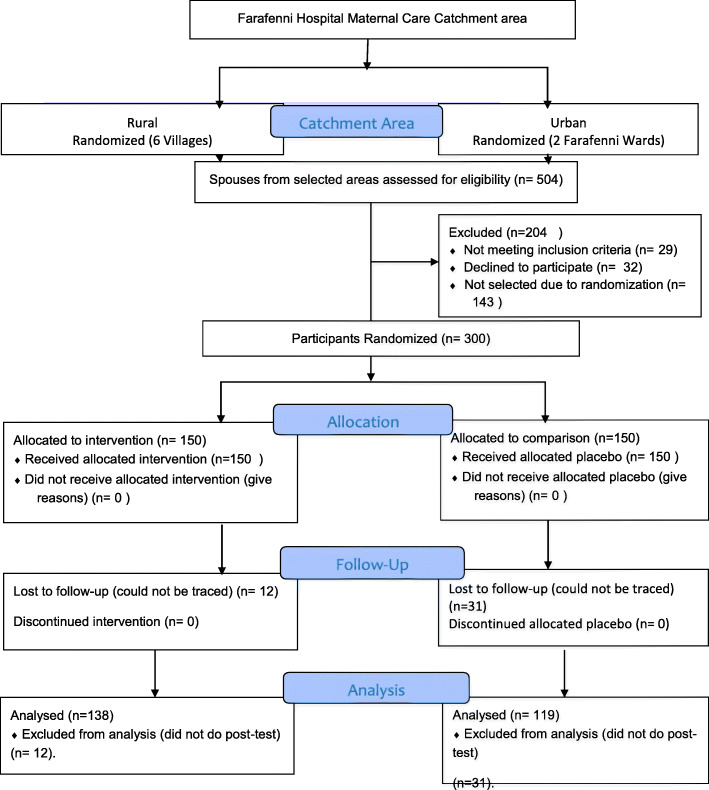
Enrollment Process

The recruitment period started from 1st October 2017 to 31st January 2018. Women from the selected six villages and Farafenni town wards meeting the gestational age criteria (they were sent for a pelvic ultrasound to confirm their gestational ages) were identified and informed about the purpose of the study during antenatal booking. The verbal consent of these women to share their gestational age information with their husbands and to allow them to participate in their obstetric care was sought before sending participation invitation letters to their husbands. This gave the women freedom to choose whether they wanted their husbands to participate in their health care or not. Five hundred and four (504) spouses of the women were assessed for the eligibility criteria but 61 of them were excluded (29 did not meet the inclusion criteria and 32 of the women did not agree with their spouses to participate). The remaining 443 spouses were sent invitation letters but 26 of them rejected the invitation and were replaced randomly. The participants’ enrolment was conducted at the antenatal clinic of Farafenni Hospital. Three hundred (300) of them were randomly selected as the study participants using the balloting with replacement method. The selected spouses from the villages and Farafenni ward identified as the intervention group’s residents were enrolled as the intervention group, while those from the comparison’s residents were the comparison group. All the selected spouses attended the first sessions but 21 (12 from comparison and 9 from intervention groups) of them did not return for the second sessions and were randomly replaced until the required sample size was achieved in both groups (see Fig. 3 for detail). The selected participants were given a detailed description of the activities, benefits, and risks involved in participating in the study.

The Principal Investigator was responsible for the randomization but the research assistants enrolled the study participants and assigned them to the intervention and comparison groups. It was only the study participants who were blinded. Blue stickers with the study identification number of the participants were pasted on the antenatal cards of the wives of spouses in the comparison, whilst green coloured stickers were for those in the intervention group. However, they were not informed of the group that they belong and participants from the intervention and comparison groups were not living together, that is, they came from different villages and Farafenni town wards. In addition, participants of the intervention and comparison groups were invited to participate in the study on different days so that they do not meet to prevent contamination of the intervention.

### Data entry and quality control

All the completed questionnaires were reviewed for completeness and incompletely filled questionnaires either of the pre-test, post-test, or both were excluded from the study. The data from the completed questionnaires were then entered into an Excel spreadsheet and scanned for errors before exporting to Statistical Package for Social Sciences (SPSS). The data was cleaned by running frequencies of all variables to check for incorrect coding. After double-checking with raw data, needed corrections were made before the analysis.

### Statistical methods

IBM Statistical Package for Social Sciences (SPSS) version 21 software was used for data analysis. Descriptive statistics were used to summarize the socio-demographic and outcome variables. The differences in the socio-demographic variables between those in the intervention and comparison groups were examined using either independent-samples t-test (for continuous variables), or chi-square (for nominal/grouped variables).

The proportion of participants that gave a correct answer to each item on the obstetric danger signs and birth preparedness indicators during the pre-and post-tests were calculated by representing the sum of the correct answers as a percentage of the total. The mean knowledge score differences between the pre-tests and post-tests of both groups (i.e., between-groups differences) were tested using the independent sample t-test and at a statistical significance level of *p* <  0.05).

Spousal level of participation in birth preparedness was analyzed by assigning a score of **1** to each indicator of the birth preparedness items that each participant reported participating in or **0** if he did not participate. The percentage of participants that reported participating in each item during the pre- and post-tests was calculated. The percentage differences in performing each item between the pre-tests and post-tests of both groups (i.e., between-groups differences) were tested using Chi-square and at a statistical significance level of *p* <  0.05).

The effect of the health education intervention on spousal knowledge and participation in birth preparedness was tested using analyses of covariance (ANCOVA) and hierarchical linear regression (to control for the demographic variables). Due to the clustering in the multistage sampling used in the study, analysis of covariance (ANCOVA) was used to adjust for any preexisting between-group differences not controlled for at the study’s onset. ANCOVA, using the pre-test mean scores as a covariate, would help to determine whether the adjusted post-test mean scores between the two sample groupings were significantly different from another (F-value; confidence level of *p* <  0.05).

Levene’s test was used to test the ANCOVA assumption of homogeneity of variance of the dependent variable. The categorical independent variables (health education and demographic variables) used as predictors of the outcome variables were transformed to dummy variables before conducting the regression analysis. The assumptions of hierarchal linear regression analysis of linearity between independent and outcome variables, normal distribution of outcome variables, and multicollinearity between the independent variables were checked and met. Principal Component Analysis was conducted to check for variability between the independent variables. Screeplot was developed and Kaiser Criterion was used to select the independent variables with Eigenvalue > 1 as predictors of the outcome variable in the hierarchical regression analysis. The statistical significance level was set at *p* <  0.05.

## Results

### Socio-demographic characteristics of participants

In the study, 150 spouses of pregnant women, who met the eligibility criteria, were enrolled as the intervention group, interviewed during the pre-test, and participated in the intervention. There was a low attrition rate of 8% in the post-test resulting in 138 interviews. An equal number of participants (*n* = 150) were enrolled in the comparison group, and 119 (79%) continued until the post-test phase of the study. The attrition rate of the comparison group was 21%. The total attrition rate of the study was 14%. However, most of the attritions were from the participants that came from the rural areas which may due to the distance they had to travel; they formed 57% (*n* = 30) of the attrition.

As shown in Table 2, the mean age of the spouses in the intervention group was 35.9 years (SD = 9.5), and 38.2 years (SD = 11.1) for those in the comparison group. The mean gestational age of their wives at booking was 24 weeks. Sixty-four percent (*n* = 88) and 67% (*n* = 80) of those from the intervention and comparison groups respectively reported that they had informal Arabic education. Most of the study participants in the intervention (*n* = 72, 52.2%) and comparison groups (*n* = 65; 54.6%) were from the urban areas. There was no statistically significant difference between the demographic characteristics of participants in the intervention and comparison groups except for the highest level of education attained (χ^2^ = 17.937, *p* <  0.05).

**Table 2:** Socio-demographic characteristics of participants in intervention and comparison groups

Demographic variables	Intervention (*n* = 138)	Comparison (*n* = 119)	Test statistics
Age in years [M (SD)]	35.9 (9.5)	38.2 (11.1)	t = 1.718, *p* = 0.087
Age of last child in years [M (SD)]	2.0 (1.4)	2.1 (1.3)	t = 1.081, *p* = 0.281
Gestational age of wife at booking in weeks [M (SD)]	24.2 (2.8)	24.4 (2.6)	t = 1.683, *p* = 0.092
**Number (percentage)**			
Place of residence			χ^2^ = 0.154, *p* = 0.695
Urban	72 (52.2)	65 (54.6)	
Rural	66 (47.8)	54 (45.4)	
Tribe			χ^2^ = 1.695, *p* = 0.792
Mandinka	28 (20.3)	30 (25.2)	
Wollof	65 (47.1)	53 (44.5)	
Fulla	39 (28.3)	32 (26.9)	
Jola	1 (0.7)	0 (0.0)	
Others	5 (3.6)	4 (3.4)	
Type of work			χ^2^ = 0.070, *p* = 0.995
Not working	3 (2.2)	3 (2.5)	
Farmer	41 (29.7)	34 (28.6)	
Civil servant	29 (21.0)	25 (21.0)	
Others	65 (47.1)	57 (47.9)	
Highest level of education			χ^2^ = 17.937, *p* = 0.022*
None	5 (3.6)	3 (2.5)	
Basic	19 (13.8)	9 (7.5)	
Senior secondary	25(18.2)	15 (12.6)	
Tertiary	1 (0.7)	12 (10.1)	
Informal Arabic (dara)	88 (63.8)	80 (67.2)	
Number of wives			χ^2^ = 0.560, *p* = 0.905
One	93 (67.5)	81 (68.1)	
Two	38 (27.5)	31 (26.1)	
Three or more	7 (5.0)	7 (5.8)	
Number of children			χ^2^ = 3.321, *p* = 0.651
None	26 (18.8)	21 (17.6)	
One	16 (11.6)	21 (17.6)	
Two	19 (13.8)	10 (8.4)	
Three	27 (19.6	24 (20.2)	
Four or more	50 (36.2)	43 (36.2)	
Place of delivery of the last child			χ^2^ = 0.080, *p* = 0.961
No child yet	26 (18.8)	21 (17.6)	
Health facility	102 (74.0)	89 (74.8)	
Home	10 (7.2)	(7.6)	

### Spousal knowledge of obstetric danger signs and birth preparedness

Table 3 shows the pre-test and post-test mean scores of participants in the intervention, and comparison groups. The comparison group had significantly higher mean scores on knowledge of danger signs of pregnancy, (t = 2.383, *p* <  0.05) and labour, and delivery (t = 3.855, *p* <  0.01) during the pre-test than those in the intervention group. However, the participants in the intervention had substantial large mean gains after exposure to the health education, while those in the comparison group recorded irrelevant differences in mean scores between the pre- and post-tests making their differences on all the knowledge indicators statistically significant (*p* < 0.001).

**Table 3:** Differences in mean scores on knowledge of obstetric danger signs and birth preparedness between intervention and comparison groups

Variable	Pre-test Mean (SD)		Test of difference	Post-test Mean (SD)		Test of difference
	Comparison	Intervention	t-test	Comparison	Intervention	t-test
Knowledge of danger signs of pregnancy	29.2 (7.3)	24.1 (6.8)	2.383*	29.5 (6.9)	58.1 (6.5)	14.315***
Knowledge of danger signs of labour and delivery	33.2 (7.9)	23.3 (7.1)	3.855**	34.9 (7.8)	66.5 (6.3)	13.506***
Knowledge on birth preparedness	29.1 (6.9)	33.5 (7.6)	1.648	29.4 (5.6)	68.0 (5.1)	18.162***
Overall knowledge	30.5 (7.4)	26.9 (7.1)	1.797	31.3 (6.8)	64.2 (5.9)	19.393***

* *p* < 0.05; **p < 0.01; ***p < 0.001

### Spousal participation in birth preparedness

As presented in Table 4, more spouses in the comparison group (*n* = 64, 53.8%) reported that they were prepared for the delivery of their wives during the pre-test as compared to those in the intervention group (*n* = 57, 41.3%). There was also a significantly higher number of spouses in the comparison group reporting that they saved money in preparation for the delivery of their wives than those in the intervention group during the pre-test (χ^2^ = 5.138; *p* < 0.05). The total levels of birth preparedness among spouses in both the intervention and comparison groups during the pre-test were rated as poor.

**Table 4:** Differences in spousal participation in birth preparedness between intervention and comparison groups

Birth Preparedness Indicators	Pre-test		Test of difference	Post-test		Test of difference
	Comparison (*n* = 119)	Intervention (*n* = 138)		Comparison (*n =* 119)	Intervention (*n =* 138)	
	n (%)	n (%)	χ^2^	n (%)	n (%)	χ^2^
Prepared for wife’s delivery	64(53.8)	57 (41.3)	3.993*	73 (61.8)	135 (97.8)	55.112***
Secured Transport	26 (21.8)	27 (19.6)	0.204	30 (25.2)	125 (90.6)	114.072***
Saved money	37 (31.1)	26 (18.8)	5.183*	55 (46.2)	127 (92.0)	64.887***
Donated blood	0 (0.0)	0 (0.0)	____	1 (0.8)	41 (29.7)	38.995***
Identified health facility	9 (7.6)	9 (6.5)	0.106	6 (5.0)	70 (50.7)	64.028***
Planned for emergency	5 (4.2)	4 (2.9)	0.321	5 (4.2)	43 (31.2)	34.215****
Bought required materials for a clean delivery	8 (6.7)	7 (5.1)	0.317	8 (6.7)	123 (89.1)	173.641***
Level of participation			3.536			187.879***
Poor	94 (79.0)	110 (79.7)		94 (79.0)	2 (1.4)	
Moderate	23 (19.3)	23 (16.7)		24 (20.2)	35 (25.4)	
High	2 (1.7)	5 (3.6		1 (0.8)	101 (73.2)	

*P < 0.05; ***p < 0.001; ____ chi-square not calculated because there is a zero value for “yes, donated blood” in both groups

In the comparison group, it was noted that spousal participation in birth preparedness was still low during the post-test. In contrast, greater improvements in participation in birth preparedness were noted in the intervention during the post-test. The majority of the spouses in the intervention group reported that they had prepared for the delivery of their wives (*n* = 135, 97.8%) by saving money (*n* = 127, 92%), securing transport (*n* = 125, 90.6%), and acquired the required materials for clean delivery (*n* = 123, 89.1%). The least performed activity of birth preparedness among the intervention was blood donation (*n* = 41, 29.7%).

### Effect of health education on spousal knowledge of birth preparedness

The ANCOVA results (Table 5) show that the participants exposed to the Health Education had a higher mean score (mean = 64.2; SD = 5.9) in their post-test, which is an indicator of better knowledge on birth preparedness (including obstetric danger signs) than those in the comparison group (mean = 31.3; SD = 6.8). After controlling for the covariate (pretest of both groups), the intervention administered to the intervention group effectively increased knowledge on birth preparedness (F (1, 255) = 376.108, *p* < 0.001).

**Table 5:** ANCOVA on the effect of health education on spousal knowledge of birth preparedness (*n* = 257)

Group	Mean (SD)	Type III Sum of Squares	df	Mean Square	f-value	*p*-value
Intervention	64.2 (5.9)					
Comparison	31.3 (6.8)					
Source						
Corrected Model		73,967.128^a^	2	36,983.564	217.672	< 0.001
Intercept		62,999.330	1	62,999.330	370.791	< 0.001
Covariate (pre-test)		21,782.492	1	21,782.492	128.204	< 0.001
Intervention group		61,364.550	1	61,364.550	376.108	< 0.001
Error		43,155.893	255	169.905		
Total		634,408.172	257	36,983.564		
Corrected Total		117,123.022	256	62,999.330		

*R*^*2*^ = 0.612; Adjusted *R*^*2*^ = 0.604

### Effect of health education on spousal participation in birth preparedness

The ANCOVA test results show that the participants exposed to health education had higher a mean score (mean = 4.4, SD = 0.5) than those in the comparison group (mean = 0.9, SD = 0.8). This shows that the spouses in the intervention group reported performing four of the birth preparedness indicators after being exposed to the intervention, as compared to those in the control group performing only one of them. From this result, one can conclude that the health intervention administered to the spouses in the intervention group had a statistically significant positive effect on spousal participation in birth preparedness (F (1, 255) = 522.414, *p* < 0.001).

### Health education and demographic variables predicting spousal level of knowledge of obstetric danger signs and birth preparedness

To further confirm the effect of the health education intervention on spousal knowledge of birth preparedness (including obstetric danger signs) after controlling for demographic variables, hierarchical linear regression was computed and the result is presented in Table 6. When the health education intervention was entered alone, it significantly predicted 60% (*R*^*2*^ = 0.596, F (1, 255 = 376.108, *p* < 0.001) of the variation in knowledge on birth preparedness between the intervention and comparison groups during the post-test. Combining the health education and the socio-demographic variables as in model 2, improved the prediction to 65% (*R*^*2*^ = 0.646, F (1, 255 = 17.656, *p* < 0.001). However, having a “secondary school education” was the only significant contributor to this prediction (β = 0.137, *p* < 0.05). There was a significant positive correlation between the level of knowledge on birth preparedness and the health education intervention (β = 0.789, *p <* 0.001).

**Table 6:** Health education and socio-demographic variables predicting knowledge on birth preparedness (*n* = 257)

Variables	Model 1			Model 2		
	B	SEB	β	B	SEB	β
Health Education						
Control (REF)						
Intervention	32.917	1.697	0.772***	33.659	1.770	0.789***
Age of participant				−0.209	0.142	−0.102
Gestational age of wife				1.276	1.531	0.116
Number of wives				−1.393	1.860	−0.041
Number of children				−1.464	0.928	−0.125
Highest level of education						
None				−1.343	5.779	−0.010
Informal Arabic				−3.127	4.651	−0.030
Basic				8.212	11.570	0.041
Secondary				8.060	3.380	0.137*
Tertiary (REF)						
Place of residence						
Urban (REF)						
Rural				−3.112	1.880	−0.073
Tribe						
Mandinka				−3.905	5.546	−0.077
Wollof				3.712	5.211	−0.087
Fulla				−3.522	5.258	−0.074
Jola				2.395	4.253	0.007
Others (REF)						
Type of work						
Not working				14.025	8.260	0.100
Farmer				−0.986	2.163	−0.021
Civil servant				0.134	3.286	0.003
Others (REF)						
Place of delivery of last child						
No child yet (REF)						
Health facility				4.458	4.024	0.091
Home				4.874	4.556	0.080
*R*^*2*^		0.5967			0.646	
F for change in *R*^*2*^		376.108***			17.656***	

*p < 0.05; ***p < 0.001

### Health education and demographic variables predicting spousal level of participation birth preparedness

Model 1 of the hierarchal linear regression analysis result presented in Table 7, shows that the health education alone was able to significantly predict 67% (*R*^*2*^ = 0.672, F (1, 255 = 522.414, *p* < 0.001) of the variation in the level of spousal participation the birth preparedness process between the intervention and comparison groups during the post-test. The combination of health education and socio-demographic variables increases the prediction to 71% (*R*^*2*^ = 0.710, F (1, 255 = 23.616, *p* < 0.001). The significant predictors among the socio-demographic variables were, “number of children the participants had” (β = − 0.152, *p* < 0.05) and attaining a “secondary school level of education” (β = 0.153, *p <* 0.05). The relationship between the health education intervention and the outcome variable was strong, positive, and significant (β = 0.820, *p* < 0.001).

**Table 7:** Health education and socio-demographic variables predicting the level of spousal participation in birth preparedness (*N* = 257)

Variables	Model 1			Model 2		
	B	SEB	β	B	SEB	β
Health Education						
No (control) REF						
Yes (intervention)	3.467	0.152	0.820***	3.396	0.159	0.803***
Age of participant				−0.017	0.013	−0.085
Gestational age of wife				0.253	0.168	0.765
Age of last child				0.048	0.082	0.031
Number of wives				−0.122	0.167	−0.036
Number of children				−0.176	0.083	−0.152*
Level of education						
None				−0.381	0.518	−0.029
Informal Arabic				0.673	0.417	0.065
Basic				0.136	0.303	0.023
Secondary				3.001	1.037	0.153*
Tertiary (REF)						
Place of residence						
Urban (REF)						
Rural				−0.083	0.169	−0.020
Tribe						
Mandinka				0.843	0.498	0.127
Wollof				1.113	0.468	0.113
Fulla				0.875	0.473	0.126
Jola				2.483	1.281	0.073
Others (REF)						
Type of work						
Not working				−0.856	0.742	− 0.061
Farmer				−0.024	0.194	−0.005
Civil servant				0.578	0.295	0.112
Others (REF)						
Place of delivery of last child						
No child yet (REF)						
Health facility				0.447	0.362	0.092
Home				−0.555	0.476	− 0.069
*R*^*2*^		0.672			0.710	
F for change in *R*^*2*^		522.414***			23.616***	

*p < 0.05; ***p < 0.001; df = 255, 254

## Discussion of study results

### Limitations of study results

The data collection method could have been affected somewhat by the twin problems of the interviewer’s bias and language barriers. To overcome these problems, the data collectors were trained thoroughly on the procedure for questionnaire administration and a professional translator was used to translate the questionnaires into the three major local languages in Farafenni (i.e., Mandinka, Fula, and Wollof). However, there remained some minor local languages in Farafenni (e.g., Serere, Manjago, Jola, Balanta, etc.). Selected participants from these minority tribes who could not understand the chosen major local languages or English and those who refused to participate were dropped out of the study and replaced. The data from study participants who did not complete the post-test were also excluded from the analysis. Therefore, the study findings are limited in information on these categories of spouses.

Only the study participants were blinded on the study groups but not the research assistants and this might have led to the interviewer’s preference. To reduce this, interviewers were instructed not to read out the lists of possible answers, but wait for the participants to answer spontaneously. Participants in either of the research groups could have received information on obstetric danger signs and birth preparedness through some other means outside the intervention carried out in this study (e.g., radio and television programmes) and such information could have accounted for the changes observed.

Although all the recruited spouses gave informed consent, provided their contact details, and participated in the pre-test and first health education sessions, some of them could not be traced during the follow-up. This was mainly because some of the participants gave incorrect addresses and phone numbers.

### Generalization of study results

The study results can only be generalized to spouses in Farafenni and its satellite villages and communities with similar characteristics to that of the study population. However, the study findings provided evidence that can help to guide the development and implementation of health education programmes aimed at improving male awareness and participation in maternal health care services in The Gambia. Secondly, this study found that even a simple antenatal educational intervention using a maximum of two contacts with men can be beneficial in promoting their participation in maternal health care. No new staff members were required and changes in staff routines were possible without increasing work hours. Thirdly, this study provided information that married men in Farafenni are educable and willing to participate in maternal health care related studies. The project shows that involving men in maternal care services in The Gambia is feasible and acceptable. Besides, the study also indicates that using men as participants in studies investigating male involvement or participation in maternal care can yield better results on male perspective than using women. Fourthly, the intervention can easily be replicated or adapted for use in similar contexts. However, because it is only possible to issue invitations if women attend health-care facilities, good intervention coverage can only be achieved where antenatal care is well attended. Elsewhere, additional community components may be necessary. Fifthly, the finding of this study has highlighted the need for tailor-made health education strategy to meet the needs of men who are not educated since the improvement in knowledge as uncovered in this study was significantly related to senior secondary school education even among the intervention group after being exposed to the health education.

### Summary of results

There was no statistically significant difference between the demographic characteristics of participants in the intervention and comparison groups except for the highest level of education attained. The comparison group had significantly higher mean scores on knowledge of danger signs of pregnancy, and labour and delivery during the pre-test than those in the intervention group. However, the participants in the intervention had substantially higher mean gains after exposure to health education, while those in the comparison group recorded irrelevant differences in mean scores between the pre- and post-tests, making their differences on all the knowledge indicators statistically significant. The health education intervention alone could significantly predict 60% (*R*^*2*^ = 0.596%, F (1, 255 = 376.108, *p* < 0.001) of the variation in knowledge on birth preparedness between the intervention and comparison groups during the post-test. Combining the health education and having a senior secondary school education to the model, improved the prediction to 65% (*R*^*2*^ = 0.646%, F (1, 255 = 17.656, *p* < 0.001). The spouses exposed to the health education were four times more likely to participate in the birth preparedness process of their wives than those in the comparison group. The most frequently reported birth preparedness indicators were saving money and identifying transport whilst the least was blood donation.

### Interpretation of results

#### Knowledge on obstetric danger signs and birth preparedness

The low level of knowledge of obstetric danger signs and birth preparedness found in the comparison group and baseline in this study is in line with what is reported in the literature. Sekoni and Owoaje (2014) reported that the majority of men from Southwest Nigeria had poor knowledge about danger signs in pregnancy [17]. It was also reported that married men in Edo State, Nigeria, had a moderate increase in knowledge of the causes of maternal mortality after been exposed to a health education session [18]. Men from Northwest Ethiopia were able to state fewer possible danger signs in the postpartum period as compared to other periods (antepartum and delivery) [19]. According to August et al.*,* 57% of the men from rural Tanzania were not knowledgeable about the danger signs in pregnancy and delivery, and could not identify the requirements for birth preparedness during the baseline of their study [20].

However, the study results demonstrated that the health education intervention had led to a significant improvement in the levels of knowledge of the obstetric danger signs and birth preparedness among the spouses in the intervention group during the post-intervention phase as compared to those in the comparison group and the baseline. The health education intervention had more predictive power on knowledge of obstetric danger signs than the demographic variables (60% versus 5%). Even though there was an increase in knowledge in some of the danger signs among the comparison group from the pre-test to post-test, but the increase among the intervention group was stronger. The most common danger signs mentioned by spouses in both groups were severe vaginal bleeding, severe headache, high-grade fever, swollen face, and swollen hands and/or feet. The most well-recognized birth preparedness indicators were saving money and identifying transport. The five common causes of maternal mortalities in The Gambia include haemorrhage, sepsis, and eclampsia [3]. Therefore, spousal awareness of their signs and the importance of saving money and identifying transport during birth plans can become useful during times of emergency when decisions must be taken and on time. Similar findings were reported in a study on the effect of a community intervention on the utilization of maternal health care in South-west Ethiopia, that the most known obstetric danger signs were vaginal bleeding, fever, and headache [21]. Pregnancy-related vaginal bleeding was the most familiar danger sign recognized by the men in a study conducted in Burayu Town, Ethiopia [22]. Mullany and colleagues also conducted a study in Nepal and reported that the couples had increased knowledge of danger signs of pregnancy as compared to those in the control group [23]. However, this current study achieved a higher percentage increase in knowledge during the post-intervention than in the Ethiopian and Nepalese studies. The significant increase in knowledge in the intervention group of this study may be associated with the fact that the health education sessions were conducted using one-to-one lecture-discussion and known samples were used as visual aids to promote understanding of the concepts taught. This helped in promoting recall of the information given and hence higher scores in knowledge.

#### Participation in birth preparedness

The study results showed that the health education intervention was able to effectively increase the level of spousal participation in birth preparedness in the intervention group. The participants in the intervention group were four times more likely to be participative in the delivery plans of their wives. However, the participants that had fewer children, had secondary school education and were exposed to health education were more likely to be highly participative. In many Gambian families, men are the breadwinners. Therefore, the priority of the men with many children will be feeding their children rather than preparing for an additional child who is yet to be born. Also, secondary school level of education improves knowledge which may, in turn, have a positive influence on the practice of birth preparedness among the interventional spouses. There was more a than two-fold increase in the proportion of spouses in the intervention group during the post-test, reporting that they saved money, secured transport, identified health facility, made emergency plans, and acquired the required materials for clean birth in preparation for the deliveries of their wives. This indicates that the spouses from Farafenni and its satellite villages are educable and willing to participate if they are involved in maternal health care services. Similarly, a study in Indonesia found that men had increased knowledge and participation in birth preparations and delivery care after being exposed to a multimedia entertainment–education intervention programme on birth preparedness [24]. The high increase (73%) in spousal participation in birth preparedness after the intervention noted in this study is different from the 21% reported in another intervention study done in Nepal [25] and 6.2% in Northern Nigeria [26]. The possible explanation for these differences is that the baseline level of birth preparedness found in this study was higher than both of the studies it is being compared with. Secondly, the Nigerian study reported that discussion about birth preparedness between husbands and wives in Northern Nigeria was uncommon because they mostly rely on God’s protection during the delivery of their pregnant wives, and most women deliver at home, making the practice of birth preparedness unnecessary. In contrast, the majority of the spouses in this study reported that their wives delivered in a health facility. There was a high baseline institutional delivery rate; i.e., 74% of the study participants reported during the pre-test that their last children were delivered in health facilities. This high baseline institutional delivery rate may be associated with the Result-Based Funding (RBF) of the Maternal and Child Nutrition and Health Project (MCNHRP), which is jointly funded by the National Nutrition Agency, Ministry of Health and Social Welfare, and WHO. This project was pilot tested in Farafenni in 2013 and expanded to the other rural parts of The Gambia in 2015. However, with spousal participation in the birth preparedness process of their wives, this rate is expected to increase significantly.

Looking at the changes on the individual indicators of birth preparedness from pre-test to post-test in both groups, the least was on blood donation. This low number of spouses willing to donate blood in preparation for obstetric emergencies after being exposed to the intervention is a cause for concern. Anaemia and haemorrhage are among the five most common contributors to maternal mortality in The Gambia [3], and blood is not sold in the health facilities but is often donated by members of family, or community, based on emergency request. Therefore, spouses saving their blood with the health facilities’ blood banks can help to prevent the delay in emergency obstetric care interventions that may require urgent blood transfusion and help to save the lives of women and newborns especially among those from the rural part of the country. Similar to the finding of this study, Gebrehiwot, Gebregziabher & Gidey reported that only 2.1% of the men who participated in a study on husbands’ participation in birth preparedness and complication readiness in Ethiopia donated blood in preparation for emergencies that might occur during the pregnancy and delivery periods of their wives [27].

## Conclusion

This study found that the health education intervention had improved the spouses’ awareness of obstetric danger signs and birth preparedness, and enhances their participation in birth preparedness which is key in the prevention of delays in seeking and reaching health care services among pregnant women in patriarchal societies like The Gambia. Therefore, it is recommended that the health education campaigns should focus more on male participation in maternal health. It is important to involve all spouses in antenatal health education programmes on how to care for their wives during pregnancy and delivery and the possible negative outcomes of neglecting maternal health concerns. Men are educable and willing to participate in maternal health care if they are involved. However, there is a need for a tailor-made health education strategy to meet the needs of men who are not educated or who are from rural areas. Increased awareness should be created on the importance of blood donation and banking to prevent delays in blood transfusion when the need arises during pregnancy and delivery. Qualitative research with former participants would be very useful in understanding the best/least well-received aspects of this intervention.

## Supplementary Information

**Additional file 1.**

## Data Availability

The datasets used and analyzed during the current study are available from the corresponding author on reasonable request.

## Abbreviations

ANCOVA: Analysis of Covariance
ANC: Antenatal Care
DHS: Demographic Health Survey
JHPIEGO: John Hopkins Programme for International Education in Gynaecology and Obstetrics
MMR: Maternal Mortality Ratio
TBAs: Traditional Birth Attendants
UNAIDS: United Nations Programme on HIV/AIDS
UNICEF: United Nations Children’s Fund
WHO: World Health Organization
