# Supplementary material for: Effects of health education on spousal knowledge and participation in birth preparedness in Farafenni Regional Hospital, The Gambia: a randomized trial
Source: BMC Pregnancy Childbirth. 2021 Feb 12;21:129. doi: 10.1186/s12884-021-03605-y (PMC7881475; doi:10.1186/s12884-021-03605-y)
Supplement: Supplementary file 1 — Additional file 1. [file 12884_2021_3605_MOESM1_ESM.docx]

# **APPENDIX A: INTERVIEW GUIDE**

CONFIDENTIAL. INFORMATION TO BE USED FOR THIS RESEARCH PURPOSE ONLY.

**EFFECTS OF HEALTH EDUCATION ON SPOUSAL PARCITIPATION IN BIRTH PREPAREDNESS AND INSTITUTIONAL DELIVERY STUDY QUESTIONNAIRE**

| **Questionnaire No.** |  |
| --- | --- |
| **Research group** |  |

**Time Interview Started: Hour:______Minute____________**

**Interview Ended: Hour:**__________**Minute:**  _____________

| **Location Information** |  |
| --- | --- |
| **Name of Village** |  |
| **Name of Participant** |  |
| **Phone Number** |  |
| **Expected date of delivery of wife** |  |

|  | **Pretest** | **Posttest** |
| --- | --- | --- |
| **DATE** |  |  |
| **INTERVIEWER'S NAME** |  |  |
| **RESULT*** |  |  |
| **NEXT VISIT: DATE** |  |  |
| **TIME** |  |  |

*** RESULT CODES:**

| **1 = COMPLETED** | **4 = PARTICIPANT CANNOT BE FOUND** | **7 = REFUSED** |
| --- | --- | --- |
| **2 = PARTICIPANT ABSENT** | **5 = Under age** | **8 = OTHER: (SPECIFY):** |
| **3 = TIME AND DATE SET FOR** |  |  |
| **LATER** | **6 = INCOMPLETE INTERVIEW** |  |

**SECTION 1. SOCIODEMOGRAPHIC INFORMATION**

First, I would like to ask you some questions about yourself.

| Q. # | QUESTION | Response …Code | Remarks |
| --- | --- | --- | --- |
|  | How old are you? | --------------------------------- years old | Stop if under age 18, explain why and thank him |
|  | What is your tribe? | Mandinka…………………………………….1  Wollof………………………………………...2  Fulla…………………………………………..3  Jola…………………………………………...4  Manjago……………………………………...5  OTHER………………………………………7  (Specify) |  |
|  | What is your religion? | Islam………………………………...............1  Christianity…………………………………..2  OTHER ……………………………………...3  (Specify) |  |
|  | What kind of work do you do? | Not working………………………………….1  Farmer……………………………………….2  Civil Servant…………………………………3  OTHER ……………………………………...4 |  |
|  | What is your highest educational level? | None………………………………………….1  Lower Basic………………………………….2  Upper Basic………………………………….3  Senior Secondary…………………………..4  College……………………………………….5  Vocational academic……………………….6  University…………………………………….7  Dara (Arabic)………………………………..8  OTHER ……………………………………..9  (Specify) |  |
|  | How many wives do you have? | One…………………………………………..1  Two…………………………………………..2  Three…………………………………………3  Four…………………………………………..4  OTHER………………………………………5  (Specify) | If more than one wife, go to question 7  If only one wife, go to question 8 |
|  | How many of your wives are currently pregnant? | One…………………………………………..1  Two…………………………………………...2  Three…………………………………………3  Four…………………………………………..4  OTHER ……………………………………...5  (Specify) |  |
|  | How many children do you have? | None………………………………………….1  One…………………………………………...2  Two…………………………………………...3  Three…………………………………………4  Four…………………………………………..5  OTHER………………………………………6  (Specify) | If response is **not “none”**, go to question 9 and 10 |
|  | How old is your last child? | …………………… ……….. (specify whether months or years) |  |
|  | Where was your last child delivered? | Health Facility……………………………….1  Home…………………………………………2 | Go to section 2 |

**SECTION 2: KNOWLEDGE ON DANGER SIGNS OF PREGNANCY AND CHILDBIRTH**

Now I would like to ask you some questions about pregnancy and childbirth. Specifically, I am going to ask you questions about three different phases that women go through when having a child. These phases are the period of being pregnant, the period of labor and birth, and the period immediately after the birth of the child.

| **Q. #** | **Question** | **Response (Code)** | **Remarks** |
| --- | --- | --- | --- |
|  | Have you ever heard of unforeseen problems related to pregnancy or childbirth that could endanger the life of a woman? | Yes……………………………………..1  No………………………………………2 |  |
|  | In your opinion, what are some serious health problems that can occur during pregnancy that could endanger the life of a pregnant woman?  PROBE: Any others? | Bleeding 1  Severe headache 2  Blurred vision 3  Convulsions 4  Swollen hands/face 5  High fever 6  Loss of consciousness 7  Difficulty breathing 8  Severe weakness 9  Severe abdominal pain 10  Accelerated/ reduced fetal movement 11  Water breaks without labor 12  Other……………………………………………………13  (specify)  Don’t know. 0 | If the response is “**don’t know**”, go to question 14 |
|  | In your opinion, could a woman die from [this problem] any of these problems? | Yes ……………………………………………………….1  No …………………………………………………………2  Don’t know …………………………………………..3 |  |
|  | In your opinion, what are some serious health problems that can occur during labor and childbirth that could endanger the life of a pregnant woman?  PROBE: Any others? | Severe bleeding 1  Severe headache 2  Convulsions 3  High fever 4  Loss of consciousness 5  Labor lasting >12 hours 6  Placenta not delivered 30 minutes after baby 7  Other …………………………………………………….8  (specify)  Don’t know……………………………………………0 | If the response is “**don’t know**”, go to section 3 |
|  | In your opinion, could a woman die from [this problem] any of these problems? | Yes ……………………………………………………….1  No …………………………………………………………2  Don’t know …………………………………………..3 |  |

**SECTION 3: KNOWLEDGE AND PRACTICE OF BIRTH PREPAREDNESS**

Now I would like to ask you some questions about preparing for childbirth. Specifically, I am going to be asking you questions about the requirements for birth preparedness and whether you have prepared for the delivery of your wife.

| Q. # | Question | Response (Code) | Remarks |
| --- | --- | --- | --- |
|  | Have you ever heard the term “birth preparedness”? | Yes…………………………………………………………1  No …………………………………………………………2 |  |
|  | In your opinion, what are some things a man can do to prepare for his wife’s delivery? | Identify mode of transport……………………………….1  Save money………………………………………………2  Identify blood donor……………………………………...3  Identify health facility ...………………………………….4  Plan for emergency………………………………………5  Purchase or clean wrapping cloth for baby……………6  Other……………………………………………………….7  (specify)  Don’t know………………………………………………..0 |  |
|  | Have you prepared for the delivery of your wife? | Yes…………………………………………………………1  No …………………………………………………………2 | If the response is **“NO”,** go to section 4 |
|  | What have you done in preparation of your wife’s delivery? | Identified mode of transport………………………………1  Saved money……………………………………………....2  Identified blood donor……………………………………..3  Identified health facility ...………………………………....4  Planned for emergency……………………………………5  Purchased or selected clean wrapping cloth for baby…6  Other………………………………………………………...7  (specify) |  |
